# Generalized perforating granuloma annulare appearing during pregnancy

**DOI:** 10.1097/JW9.0000000000000041

**Published:** 2022-08-15

**Authors:** Christine E. de Guia, Patricia Anne T. Tinio

**Affiliations:** Department of Dermatology, Rizal Medical Center, Pasig City, Philippines

## Dear Editors,

Granuloma annulare (GA) is a benign granulomatous dermatosis with a prevalence of 0.1% to 0.4%. Generalized perforating GA is an extremely rare variant.^1^

**Fig. 1.**
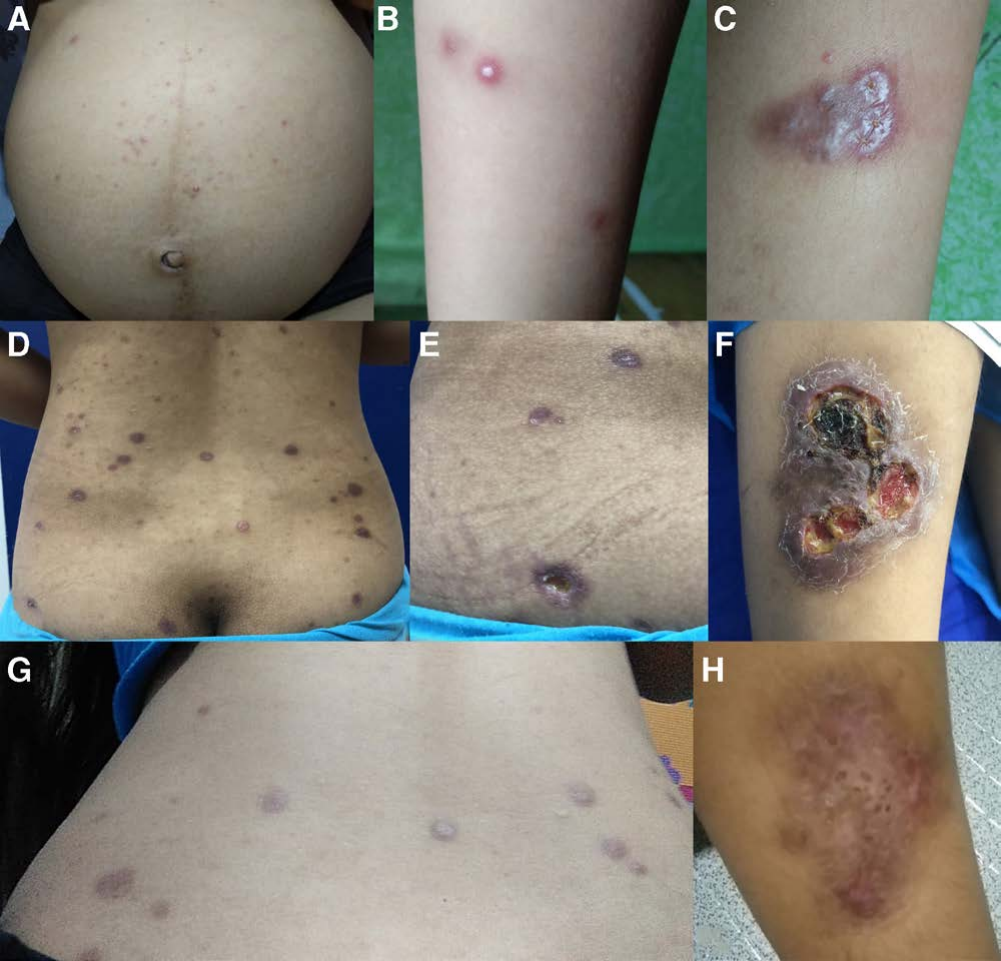
(A–C) On the 37th week age of gestation, multiple erythematous papules and plaques developed, some with central punctum as well as overlying vesicles and pustules; (D–F) on day 6 postpartum, multiple erythematous to hyperpigmented papules and nodules developed on the torso and extremities, some perforated, with an ulcerated plaque with yellowish discharge and black crusts on the right lower leg; (G, H) On day 49 postpartum, after 2 wk on topical steroids, further flattening of lesions and healing of perforated areas were noted.

**Fig. 2.**
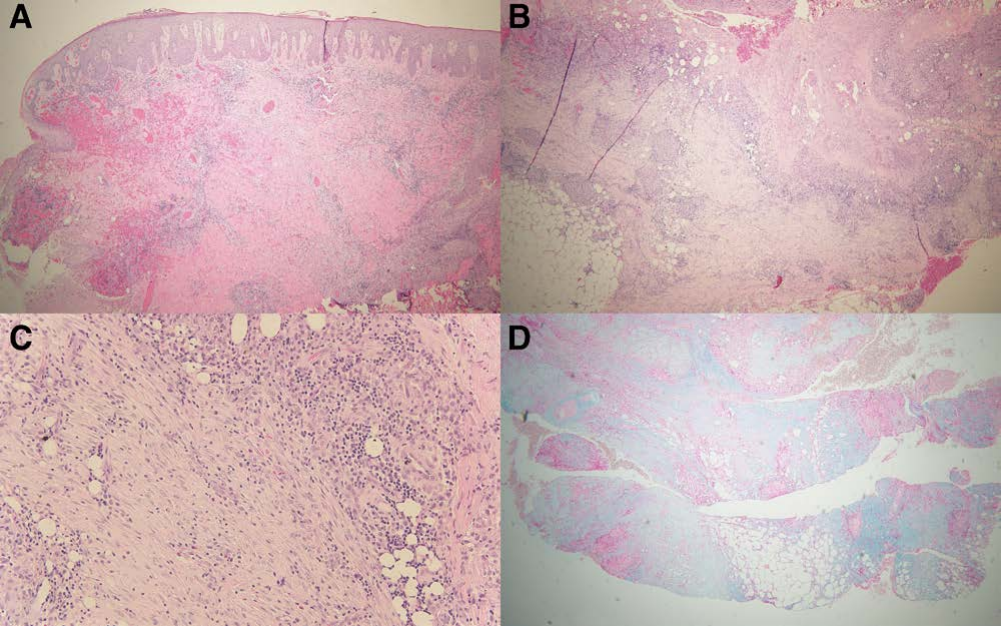
(A–C) The incisional wedge biopsy revealed parakeratosis, moderate spongiosis with pseudoepitheliomatous hyperplasia and areas of focal ulceration and follicular involvement, palisading granulomas around small foci of mild connective tissue degeneration with mucin accumulation, superficial and deep moderate infiltrates of lymphocytes, neutrophils, eosinophils, and plasma cells, as well as deep dermal scarring; (D) Alcian blue stain confirmed the presence of mucin.

We report a case of a 20-year-old G2P1 (T1-P0-A0-L1) who presented with multiple pruritic papules on the legs and trunk during her first trimester, with no systemic symptoms. She was not on any medication and has no known illnesses, with no similar symptoms during her first pregnancy. On her 37th week age of gestation, the lesions developed central punctum and overlying vesicles and pustules (Fig. 1A–C). Differential diagnoses at this time were papular urticaria, pemphigoid gestationis, and polymorphic eruption of pregnancy. On day 6 postpartum, her lesions evolved into violaceous papules and plaques, some ulcerated with discharge and tenderness (Fig. 1D–F). At this time, generalized pyoderma gangrenosum was considered. Wound gram stain and culture showed negative results. Complete blood count, urinalysis, liver function, and fasting blood sugar were normal. On day 20 postpartum, an incisional wedge biopsy was done on the lesion in Figure 1F, revealing findings consistent with perforating GA (Fig. 2).

On day 35 postpartum, the patient noted improvement and resolution of pain even without treatment. She was prescribed clobetasol propionate ointment twice daily for 2 weeks, after which she reported further improvement and healing of perforated areas (Fig. 1G, H). At 3 months postpartum, continuous improvement with no recurrence was noted.

Generalized perforating GA is rarely reported in literature. It is seen in patients aged 1 to 60 years, predominantly females.^1^ The etiology of GA is unclear, although it is hypothesized to be an immune reaction of the body inciting a proinflammatory state leading to tissue destruction.^2^ Although majority of cases are idiopathic, triggers have been reported for generalized perforating GA, including insect bites, ultraviolet radiation, trauma, thyroiditis, and diabetes mellitus.^1^

In our patient, there were no apparent triggers or associated illnesses based on a thorough history and laboratory tests. It is noteworthy that she was pregnant when her lesions appeared, and improvement even without treatment occurred at about 1 month postpartum. To our knowledge, there are no studies in literature demonstrating an association between GA and pregnancy. One study reported a pregnant patient with GA whose lesions resolved at the fifth month of pregnancy, but returned at 6 months postpartum and persisted for 6 years. Another patient in this study noted recurrence of lesions during pregnancy, but resolution after 3 months while still pregnant.^3^ During pregnancy, the immune system undergoes complex changes that alters one’s inflammatory response. Particularly, the first and last trimesters are characterized as proinflammatory states, with high levels of T-helper 1 cells and its associated cytokines.^4^ Although the etiology of GA is not fully elucidated, several studies support a delayed Th1 immune response, with Th1-associated cytokines inducing inflammation and subsequent tissue destruction in the skin, as evidenced by the characteristic symptoms and histopathology in GA.^2^ These similarities in the immunologic profiles of both pregnancy and GA have led us to hypothesize that immune system changes during pregnancy may have contributed or possibly triggered GA in our patient, especially considering that generalized GA responds poorly to treatment,^5^ but her lesions improved postpartum even without intervention.

Although no established association exists, pregnancy is a condition that warrants further investigation as a possible triggering or associated condition in GA, as our patient’s lesions coincided with pregnancy and resolved postpartum.

## Conflicts of Interest

None.

## Declaration of patient consent:

Informed, written consent was received from all patients and confirmed to the journal pre-publication, stating that the patients gave consent for their photos and case history to be published.

## Funding

None.

## Study Approval

N/A.
